# Supplementary material for: Are carbon emissions trading and green financial instruments synergistic? -Comprehensive quantitative research based on content analysis
Source: PLoS One. 2024 Mar 7;19(3):e0298601. doi: 10.1371/journal.pone.0298601 (PMC10919601; doi:10.1371/journal.pone.0298601)
Supplement: S1 Data — (ZIP) [file pone.0298601.s001.zip › Data/地级市Z_F工作报告.zip!/╣π╬≈/░┘╔1⁄2╩╨/└┤╫╘╣1⁄2╓┌║┼í╛┬φ┐╦╩2╛▌═°í┐_2012.docx]

各位代表：

　　现在，我代表市人民政府向大会报告政府工作，请予审议，并请市政协委员和其他列席会议的同志提出意见。

　　一、2011年工作回顾

　　过去一年，是我市“十二五”良好开局之年，是经济社会实现新发展的一年。面对复杂多变的发展形势，在市委的坚强领导下，我们深入贯彻落实科学发展观，加快转变经济发展方式和经济结构调整，努力克服缺电、缺水、缺资金等重重困难，强化措施，克难攻坚，保持了经济社会平稳较快发展的良好势头，开创了改革开放和现代化建设的新局面。预计，2011年地区生产总值达680亿元，增长10%；全社会固定资产投资达760亿元，增长18.9%；财政收入达84亿元，增长16.2%，首次实现全市12个县（区）全部进入财政收入超亿元县（区）行列，平果县、田东县、靖西县财政收入超10亿元；社会消费品零售总额实现134.6亿元，增长18%；城镇登记失业率3.3%，低于4%的控制目标；城镇居民可支配收入17570元，增长10%；农民人均纯收入4155元，增长20%，是撤地设市以来增长最快的一年。

　　（一）投资和项目建设取得新突破。全市统筹推进重大项目912项，比上年增加232项，投资规模再创历史新高。田东石化总厂年产60万吨重油催化裂化联合装置、中铝广西分公司年产220万吨赤泥选铁、广西绿实环保材料公司聚合氯化铝、那比水电站等项目竣工投产；云桂铁路、百色至靖西高速公路、鱼梁航运枢纽工程、广银铝业一期年产20万吨铝材加工、田东锦鑫化工年产100万吨多品种化学铝等项目加快推进；瓦村水电站、金麦克生物科技公司年产100万吨生物蛋白饲料和生物菌肥等项目开工建设。

　　（二）经济结构调整取得新进展。三次产业结构由上年18.3：54.7：27.0调整为17.6：55.2：27.2，经济的整体质量得到进一步提高。

　　工业化进程加快推进。全市规模以上工业总产值完成700亿元，增长22%；工业增加值完成266亿元，增长11%。“千亿铝产业”工程取得新进展，铝加工产量增长20.3%；铝及配套产业实现工业产值348亿元，占全市规模以上工业的49.7%。百色新山铝产业示范园等园区建设加快推进，全市工业园区完成工业增加值217.8亿元，增长13%。平果工业园区和靖西铝工业园区进入百亿元园区行列，全市百亿元园区达3个。广西百色生态型铝产业示范基地获得国家批准设立，并纳入全区发展战略。39家中小企业入选自治区“千家成长型中小企业”计划。

　　现代农业加快发展。全市粮食产量108万吨，增长3.8%。“南菜北运”生产基地规模不断壮大，全市蔬菜种植面积达163万亩，产量277.1万吨，分别增长6.3%和9.8%。水果、糖料蔗、鲜茶、鲜茧、食用菌、中草药、烟叶、肉类、水产品产量稳定增长。林下养鸡“千百十万”工程加快实施，全市肉鸡出栏5150万羽，完成全年任务的102%。“百万亩竹子产业基地”建设加快，新种竹子16.5万亩，总面积达67.7万亩。林下种植面积达18.2万亩。实现国家级重点龙头企业零的突破，新增自治区级重点龙头企业2家、市级9家，2个村被评为全国“一村一品”示范村。新增农民专业合作经济组织115家。全市无公害农产品、绿色食品和有机农产品基地认证总面积达197.2万亩，有机农产品面积居全区第一。乐业县获准创建国家有机农业示范县。

　　第三产业蓬勃发展。初步建立现代服务业基本框架。红色旅游品牌影响力不断扩大，游客人数再创历史新高，全市接待旅游总人数、综合收入分别增长16.2%和29.7%。平孟口岸获批为国家一类口岸。中国——东盟(百色)农业新技术新品种交易中心等一批物流项目开工建设和竣工。公路货运周转量增长38.5%。第三产业增加值达185亿元，增长9%。

　　新兴产业加快培育。生物科技、新材料造纸、竹子加工等新兴产业逐步兴起。铝基新材料、非冶金级氧化铝等产业加快谋划布局。有机农业加快发展。煤矿安全装备制造和科技研发取得重要进展，桂林电子科技大学百色科技产业孵化园、美联能源科技产业园竣工落成。

　　（三）城镇面貌取得新变化。城镇化率达到35.5%，比上年增长2个百分点。全市建设领域完成投资154.4亿元，增长18.8%。撤地设市10周年城建重点工程取得新进展，竹洲大桥、百林大桥、竹洲大道、火车站站前大道东段、东环大道等城市环路建成通车，市体育中心、龙旺大桥等一批项目开工建设，东合大桥、市科技文化中心等项目加快推进。市区新区主骨架路网基本形成，50平方公里的市区新区框架显现。

　　（四）改革开放迈出新步伐。“农事村办”、“农情乡解”改革进一步深化拓展。集体林权制度主体改革提前两年完成。金融改革取得新突破，全市村镇银行达到4家，成为全区村镇银行最多的市；田东县成为全国首个“信用县”。率先在全区推行市级价格调节基金制度和临时价格补贴政策。医药卫生体制五项重点改革加快推进，基层医疗机构实现国家基本药物制度和卫生综合改革全覆盖。公立医院改革试点工作启动。文化体制改革稳步推进。自治区直管县财政体制改革加快推进。电网股份制改革工作稳步推进。开放合作战略深入实施，成功举办第四届中国——东盟（百色）现代农业展示交易会，与越南河江省、高平省分别签署全面合作框架协议和建立友好合作关系备忘录，中国龙邦——越南茶岭跨境经济合作区前期工作顺利启动。

　　（五）生态文明建设取得新进步。生态文明示范市建设步伐加快，“绿满百色”造林绿化工程和“右江森林河谷”创建工作不断推进。尾矿、废矿及冶炼废渣循环综合利用和铝土矿区生态复垦稳步推进。7个农村环境连片整治示范项目建设进展顺利。国家石漠化综合治理工程、大石山区石漠化综合治理与扶贫开发试点工作扎实推进。城镇饮用水源保护区划定工作进展顺利。万元生产总值能耗下降4%，化学需氧量下降4%、二氧化硫排放量下降15.5%、氨氮排放量下降7.5%，氮氧化物减排控制在国家许可范围之内。

　　（六）民生工作取得新成效。全市财政对民生投入达113.2亿元，占财政总支出的69.3%，增长26.3%。城镇新增就业1.96万人，农村劳动力转移就业新增7.43万人。养老、医疗、失业、工伤和生育保险参保人数达95.29万人次，增长7.9%。9个县（区）城乡居民社会养老保险试点扎实推进。52.18万人享受城乡低保补助，基本实现应保尽保。“新农合”参合率达97.63%，超过国家要求7.63个百分点。补助贫困学生生活费1.72亿元，受益学生32.56万人次。田东县、那坡县、德保县农村义务教育学生营养改善试点顺利启动。大石山区人畜饮水工程建设大会战、农田水利建设大会战和病险水库除险加固任务圆满完成，第三批291个贫困村“整村推进”通过自治区检查验收。开工建设保障性住房2.25万套，开工率达142.9%；新增廉租住房租赁补贴1000户全部补贴到位；实施农村危房改造3.03万户，竣工率达102%。对限价猪肉、牛肉、大米、食用油、蔬菜、液化气以及城镇低保、五保对象和市区公交车实行市级临时动态价格补贴，补贴资金1585万元。抗旱救灾取得全面胜利。十项为民办实事工程全面完成。

　　（七）社会各项事业实现新发展。教育布局调整规划稳步推进，学前教育三年行动计划全面实施，国家教育扶贫试点项目启动，三年职业教育攻坚工程全面完成，高考专科以上上线率97.08%，连续两年居全区第二位。全民健身运动蓬勃开展，成功举办一系列国际赛事，在广西第十二届运动会上勇创佳绩。建成87个村级公共服务中心、10个乡（镇）文化站和1128个“农家书屋”。各县（区）文化节庆活动繁荣活跃。百色壮族文化生态保护区建设稳步推进。1.02万个20户以上“村村通”广播电视工程顺利完成。1810个村卫生室建设全面完成。全国人口计生综合改革示范市建设不断深化，人口自然增长率6.64％。，控制在8％。以内。

　　（八）社会管理创新得到新提升。以生产管理机械化、信息化、标准化为核心的“百色经验”得到中央和自治区的肯定并在全国推广，道路交通、烟花爆竹、矿产资源开发等一批安全生产监督机制创新取得初步成效。煤矿职业安全健康工作迈上新台阶。农产品、食品和药品安全整治扎实开展。应急救援体系初步建立。公安机关“三项重点工作”和“三项建设”深入实施。

　　二、2012年工作的总体要求和目标任务

　　2012年是党的十八大召开之年，是实施“十二五”规划承前启后的重要一年，也是我市撤地设市10周年，做好今年的各项工作意义十分重大。

　　2012年我市经济社会发展的预期目标是：地区生产总值增长12%；全社会固定资产投资增长20%；工业总产值增长26%；财政收入增长16%；社会消费品零售总额增长17.5%；外贸进出口总额增长12%；万元地区生产总值能耗下降3.7%，二氧化碳、二氧化硫、化学需氧量、氨氮和氮氧化物减排按自治区下达指标执行；城镇居民可支配收入增长11%；农民人均纯收入增长15%；城镇登记失业率控制在4%以内；人口自然增长率控制在8％。以内；居民消费价格指数涨幅控制在5%左右。

　　实现上述目标，我们要着重抓好以下工作：

　　（一）着力保持投资和项目建设对扩大内需的强劲拉动，促进经济平稳较快发展。

　　统筹抓好重点领域投资。统筹推进工业、交通、城建、社会民生、农林水等领域项目建设，力争产业投资完成360亿元，交通基础设施投资完成150亿元，城镇基础设施投资完成145亿元，社会民生领域投资完成60亿元，水利基础设施和农林牧渔业项目完成投资30亿元，进一步扩大投资规模。

　　千方百计提高重大项目开竣工率。大力推动重大项目新开工，力争项目开工率达80%以上，完成投资330亿元。加快192个续建重大项目建设进度，完成投资130亿元。确
